# Development of Biodegradable Starch Microspheres for Intranasal Delivery

**DOI:** 10.4103/0250-474X.41450

**Published:** 2008

**Authors:** A. V. Yadav, H. H. Mote

**Affiliations:** Department of Biopharmaceutics, Government College of Pharmacy, Karad-415 124, India

**Keywords:** Microspheres, domperidone, starch, nasal drug delivery, mucoadhesion, bioadhesive, crosslinking

## Abstract

Domperidone microspheres for intranasal administration were prepared by emulsification crosslinking technique. Starch a biodegradable polymer was used in preparation of microspheres using epichlorhydrine as cross-linking agent. The formulation variables were drug concentration and polymer concentration and batch of drug free microsphere was prepared for comparisons. All the formulations were evaluated for particle size, morphological characteristics, percentage drug encapsulation, equilibrium swelling degree, percentage mucoadhesion, bioadhesive strength, and in vitro diffusion study using nasal cell. Spherical microspheres were obtained in all batches with mean diameter in the range of above 22.8 to 102.63 μm. They showed good mucoadhesive property and swelling behaviour. The in vitro release was found in the range of 73.11% to 86.21%. Concentration of both polymer and drug affect *in vitro* release of drug.

For systemic therapy, the nasal route has attracted increasing attention as a suitable method for drug delivery. The nasal cavity as a site for systemic absorption of drug has some advantages which include large surface area, porous endothelium basement membrane, highly vascularised epithelial layer, high total blood flow per cm^3^, avoids first pass metabolism and easy access. The nasal route can also be used for patients under emesis and with respect to parental route the nasal route is not invasive^1^–^3^.

The design of nasal dosage form has to consider the anatomical and physiological characteristics of nasal mucosa and more particularly the rapid mucociliary clearance that limits the time allowed for drug absorption to occur and effectively rule out sustained nasal administration. To overcome the rapid clearance and to facilitate the absorption through the barrier of nasal mucosa, mucoadhesive material and absorption promoters are used in nasal formulations^4^.

Starch was used as polymeric material. Starch is biocompatable, biodegradable and bioadhesive in nature^5^. The concept of using bioadhesive delivery system in the form of degradable starch microsphere (DSM) for nasal delivery of drug was introduced by Illum *et al*^6^. DSM is not only biodegradable, but also shows a high degree of swelling when in contact with aqueous medium. Starch forms a gel like system, with prolonged residence time in the nose and significant contact with the nasal mucosa. In addition starch microspheres do not produce immune response. Starch microspheres are most promising for nasal administration^7^.

Domperidone is a potent antiemetic^8^, antimigraine^9^ drug effective for preventing different kinds of emesis. Its conventional dosage form such as tablet and suspension give poor bioavailability (18%) due to extensive first pass effect, whereas on rapid i.v. injection it has been shown to cause cardiac arrhythmias^10^. This study investigates the design, development and evaluation of microspheres for nasal delivery.

Starch microspheres were prepared by emulsification-crosslinking technique, using epichlorohydrin as crosslinking agent. The microspheres were characterized in terms of particle size morphology, percentage yield, percentage drug encapsulation, mucoadhesive property, swelling behaviour, bioadhesive strength and *in vitro* diffusion using nasal cell.

## MATERIALS AND METHODS

Domperidone was procured as a gift sample from Torrent Pharmaceutical, Ahmedabad. Soluble starch, epichlorohydrine, cyclohexane, chloroform, span-60, ethanol, sodium hydroxide were purchased from Loba Chemie, Mumbai. All reagents used were of analytical reagent grade.

### Preparation of starch microspheres:

Cross-linked starch microspheres were prepared by emulsion-crosslinking method^11^. For a typical batch, the aqueous phase was prepared by dissolving 8 g of soluble starch in 12 g of a 2 M sodium hydroxide solution under mechanical stirring. The aqueous phase was pre-emulsified in 1000 ml of cyclohexane:chloroform mixture (4:1, v/v) containing 0.5% (v/v) of span-60. The emulsion was homogenized by high-speed mechanical stirring for 3 min. Further, a suitable amount of epichlorohydrin (2.5%) was added under magnetic stirring at 1000 rpm. The stirring was maintained for 18 h at 40º. Microspheres were isolated by centrifugation and washed twice with cyclohexane and distilled water. Finally microspheres were freeze dried and kept in a closed container. Formulation variables such as different amounts of drug concentration and concentration of polymer was evaluated to obtain the microspheres of optimum properties. Composition of variable is given in Table 1.

**TABLE 1:** FORMULATION VARIABLE FOR DIFFERENT BATCHES OF STARCH MICROSPHERES OF DOMPERIDONE

Formulation code	Soluble starch (% w/v)	Domperidone (mg)
Ds_1_	8	100
Ds_2_	8	125
Ds_3_	8	150
Ps_1_	6	100
Ps_2_	10	100
Ps_3_	12	100

### Characterization of microspheres:

Total amounts of microspheres obtained were weighed and the percentage yield calculated taking into consideration the weight of drug and polymer. The particles were grossly separated into different fractions passing through a set of a sieves and then the particle size was determined using optical microscopic method with the help of calibrated eye piece micrometer. The size of around 300 particles were measured and the average particle size determined^12^. Surface morphology of microspheres was studied using scanning election microscopy (SEM) using Hitachi (Model S-2400)^13^. Microspheres were sprinkled on to double side tape, sputter-coated with platinum and examined in the microscope at 15 kV. Encapsulation was determined by taking weighed quantity of starch microspheres (approximately 25 mg) in a 25 ml volumetric flask, sufficient quantity of ethanol was added to make 25 ml. The suspension was shaken vigorously and then left for 24 h at room temperature with intermittent shaking. Supernatant was collected by centrifugation and drug content in supernatant was determined using UV spectrophotometer at a suitable wave length (286 nm)^14^. Efficiency of drug entrapment for each batch was calculated in terms of percentage drug entrapment (PDE) as per the formula, % drug entrapment= Actual drug content/theoretical drug content ×100.

The equilibrium swelling degree (ESD) of starch microspheres was determined by swelling a suitable volume of dried microspheres in 5 ml ethanol, overnight in a measuring cylinder. The ESD (ml/g) was expressed as the ratio of the swollen volume to the mass of dried microspheres^15^.

The bioadhesive strength of all batches was determined using measuring device^16^. Section of nasal mucosa was cut from the sheep nasal cavity and instantly secure with mucosal side out on glass vial. The vial using nasal mucosa was stored at 37º for 5 min. Next, one vial with a section of mucosa was connected to the balance and the other vial was placed on a height-adjusted pan. Microspheres were placed in between the adjusted vial. The weight was increased until two vials were detached. Bioadhesive force was determined for the minimum weight that detached two vials.

Mucoadhesive properties of microspheres were studied by rat gut loop method described by Dhawan *et al*^17^. The required amount of starch microspheres were suspended in physiological saline solution and sonicated (100 mg in 5 ml). The suspension of microspheres was filled in to a loop of small intestine (~15 cm in length) of sheep and sealed. This tube was incubated in saline solution at 37º for 60 min, microspheres suspension was then removed. The adhered microspheres amount was estimated from the difference between the applied microspheres amount and the flowed microspheres amount. The ratio of the adhered microspheres to the applied microspheres was computed as percent mucoadhesion. *In vitro* nasal diffusion study was done by using nasal diffusion cell^18^, having three openings each for sampling, thermometer and donor tube chamber. The nasal mucosa of sheep was separated and it was attached to donor chamber tube. Diffusion of domperidone from microspheres was studied at 37±2º. An amount of microspheres equivalent to about 5 mg of domperidone were added on the mucosal surface at zero time. Samples were withdrawn at different time intervals upto 8 h and it was further diluted with ethanol upto 5 ml and absorbance was measured spectrophotometrically at 286 nm to evaluate the amount of drug release.

## RESULT AND DISCUSSION

Physical characteristics of microsphere are shown in Table 2. DSM prepared in all the batches were spherical in shape. In previous studies, Illum *et al*^6^, found that particle size was related to intranasal drug absorption. A size range of 40-60 mm was suitable for nasal administration. The size of starch microspheres prepared in this study was in the range of 22.8 to 102.63  µm, which is favourable for intranasal absorption. It was observed that as the amount of polymer and drug concentration increased in the microspheres the particle size also increased proportionally. The increase in the particle size observed with increase in polymer and drug concentration was due to increase in viscosity of the droplet^19^.

**TABLE 2:** PHYSICAL CHARACTERISTICS OF PREPARED MICROSPHERES OF DOMPERIDONE

Formulation code	Particle size (μm)^a^	Product yield (%)	Encapsulation efficiency (%)	Equilibrium swelling degree (ml/g)^a^	Mucoadhesion (%)^a^	Bioadhesive strength (g)
Ds_1_	22.43±1.30	56.20	26.30	3.61±0.091	82.93±1.85	7.86±0.04
Ds_2_	37.50±0.88	68.30	29.82	3.27±0.14	78.27±2.13	7.45±0.070
Ds_3_	42.70±1.34	66.25	34.46	3.18±0.17	83.81±1.7	7.31±0.050
Ps_1_	47.50±1.25	73.56	43.13	3.52±0.090	79.80±1.23	8.32±0.065
Ps_2_	62.64±2.08	79.26	45.23	4.05±0.095	74.77±1.44	8.72±0.070
Ps_3_	102.63±2.61	78.21	56.20	4.54±0.10	72.51±1.90	9.46±0.51
Plain	23.26±1.36	67.48	-	3.27±0.16	75.15±0.88	8.89±0.32

a Mean±SD

**Fig. 1:**
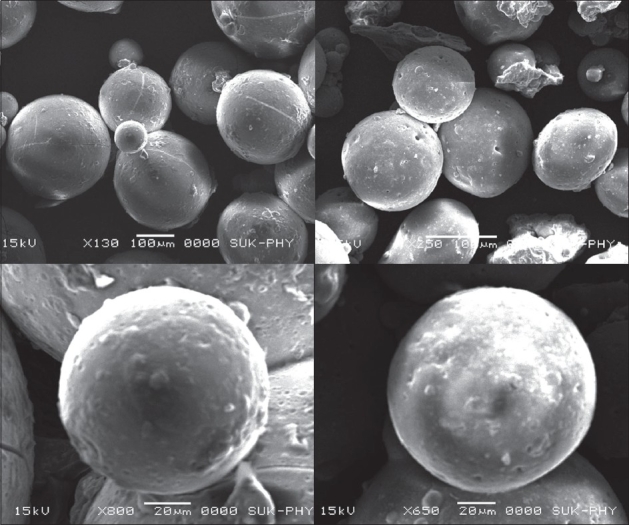
SEM Image of domperidone and domperidone-loaded microspheres. SEM Image of domperidone-free (left) and domperidone - loaded (right) microspheres prepared by emulsification - crosslinking method.

The yield of starch microspheres was obtained in range of (84-89%) SEM analysis of microspheres revealed that all microspheres prepared were spherical and smooth in shape. Fig. 1 shows morphological image of domperidone free and domperidone loaded starch microspheres. The percentage drug encapsulation was found to be 26.8% to 56.2% as shown in Table 2. It was observed that polymer and drug concentration affect PDE. Highest PDE was found (56.2%) for 10% w/v of starch. Increase in polymer concentration attributed to increase in viscosity, which resulted in formation of large droplets, thus increasing the PDE.

Equilibrium swelling degree increases as the concentration of polymer increases while it decreases as concentration of drug increases as compared to plain starch microspheres. It can be concluded that incorporation of drug in microspheres decrease ESD. ESD was found to be 3.12 to 4.63 ml/g.

Percentage mucoadhesion was found in the range from 72.4% to 83.5%. Bioadhesive strength was in range from 8.51 g to 9.67 g. Thus starch microspheres prepared were found to be having good mucoadhesive property.

**Fig. 2:**
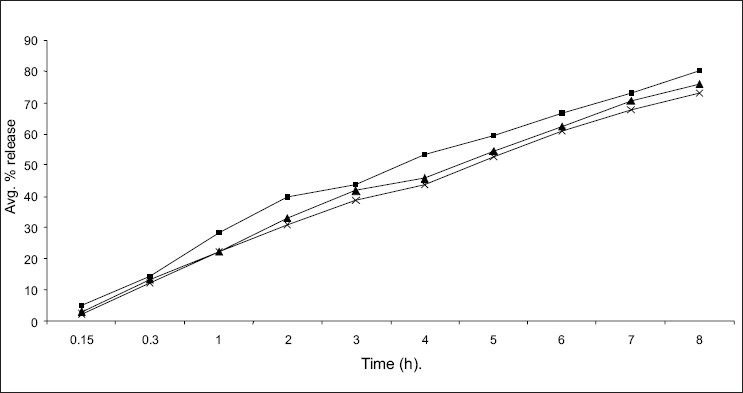
*In vitro* diffusion study of domperidone from starch microspheres containing different concentration of starch. Microspheres PS1 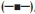, PS2 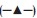 and PS 3 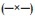 containing different concentrations of starch.

**Fig. 3:**
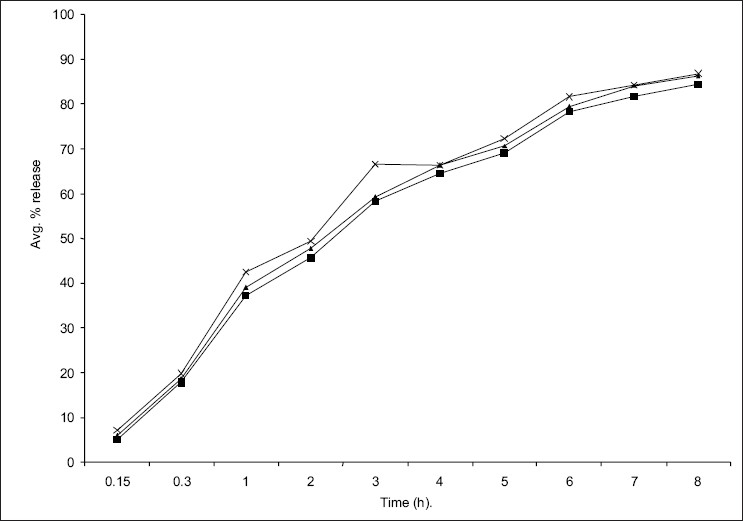
*In vitro* diffusion study of domperidone from starch microspheres containing different concentration of domperidone. Microspheres DS1 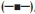, DS2 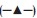 and DS3 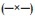 containing different concentrations of domperidone.

The *in vitro* diffusion profile of domperidone from microspheres is shown in figs 2 and 3. The release profile of formulation code Ds_3_ and Ps_3_ were highest and lowest (86.21% and 73.11%) respectively. It was observed that domperidone is released from the microspheres in sustained release manner. Formulation variables such as concentration of polymer and drug affect rate and extent of drug release, which is due to increase in density of polymer and also increase in diffusion path length that the drug molecule has to travel^20^. It is also revealed that release of drug from microspheres is directly proportional to concentration of drug. Thus sustained release of domperidone from microspheres may be achieved as a desirable property for formulation, treating conditions involving vomitting and migraine.
